# A Compact Device Model for a Piezoelectric Nano-Transistor

**DOI:** 10.3390/mi16020114

**Published:** 2025-01-21

**Authors:** L. Neil McCartney, Louise E. Crocker, Louise Wright, Ivan Rungger

**Affiliations:** National Physical Laboratory, Hampton Road, Teddington TW11 0LW, UK; louise.crocker@npl.co.uk (L.E.C.);

**Keywords:** piezoelectric, transistor, RF switch, VLSI device, multilayers, switching voltage

## Abstract

An approximate compact model was developed to provide a convenient method of exploring the initial design space when investigating the performance of micro-electronic devices such as nano-scaled piezoelectronic transistors, where fast ball-park estimates can be very helpful. First of all, the compact model was verified by comparing its predictions with those of accurate axi-symmetric finite element analysis (FEA) using special boundary and interface conditions that enable the replication of the analytical model behaviour. Verification is achieved for a radio frequency (RF) switch and a smaller very-large-scale integrated (VLSI) device, where percentage differences between the compact and FEA model predictions are of the order 10^−4^ for the RF switch and 10^−5^ for the VLSI device. This confirms the consistency of complex property data (especially electro-thermo-elastic constants) and geometrical parameter input to both types of models and convincingly demonstrates that the analytical models and FEA for the two devices have been implemented correctly. A second type of boundary and interface condition is also used that is designed to replicate the actual behaviour of the devices in practice. The boundary and interface constraints applied for the verification procedure are relaxed so that there is perfect interface bonding between layers. For this unconstrained case, the resulting deformation is very complex, involving both bending effects and edge effects arising from property mismatches between neighbouring layers. The results for the RF switch show surprisingly good agreement between the predictions of the analytical and FEA results, provided the thickness of the piezoelectric layer is not too thick, implying that the analytical model should help to reduce the parameter design space for such devices. However, for the VLSI device, our results indicate that the compact model leads to much larger errors. For such systems, the compact model is unlikely to be able to reliably reduce the parameter design space, implying that accurate FEA will then need to be used.

## 1. Introduction

Some types of micromachines can be thought of as an assembly of a number of electro-mechanical elements which are electrically or mechanically stimulated. Piezoelectric (PE) materials, enable an electrical signal to be converted into a mechanical response, and vice versa. They are often used in sensors, in actuators, or as thin films for energy harvesting, and have more recently been used as components in fast, low-power transistors responding to trends in miniaturisation and particularly with the introduction of Nano-Electro-Mechanical Systems (NEMS) that are used in many microelectronic devices. Such applications require the use of thin piezoelectric films with nanoscale through-thickness dimensions, where the film, electrodes, and insulating layers are all bonded together, and to much thicker substrates, leading to multi-layered substructures of rectangular cross-section (i.e., stacks) in which the piezoelectric layers are embedded. The electro-mechanical behaviour of such stacks has been analysed in detail in reference [1]. Sometimes (see, for example [2]), the electrodes are modelled as being infinitesimally thin to simplify analyses.

Perhaps the simplest form of a micromachine is a piezoelectronic transistor (PET), as described in the references [3,4]. The invention exploits the stress transduction operating principle whereby an applied voltage signal charges a piezoelectric (PE) capacitor, which then expands, compressing a piezoresistive (PR) channel material against a ‘rigid’ surrounding yoke, transducing an electrical input signal into mechanical stress. The PR undergoes an insulator-to-metal phase transition as a result of applying axial compressive stress, turning the device ‘on’ and thereby transducing the induced mechanical stress into an output voltage signal. The transduction of the input voltage to an induced stress state of the PET device means that the current through the PR channel is controlled by the stress-dependent resistivity of the PR material. Increased quantitative understanding of PET devices has required the use of finite element analysis (FEA), as in reference [5], whereby a radio frequency (RF) switch and a smaller PET (to be referred to as a very-large-scale integration (VLSI) device) were analysed. An RF switch is a transistor-based device to route high-frequency signals through transmission paths while transistors in VLSI devices having smaller dimensions are used when creating integrated circuits by combining very large numbers of MOS (metal oxide semiconductor) transistors on a single chip.

In addition to the geometrical complexity arising from the presence of stacks of perfectly bonded cuboidal multi-layers, 3D analysis methods can also be complex due to layer anisotropy and they are time consuming when undertaking finite element analysis. For the applications to be considered here, equi-biaxial stress and strain conditions can be assumed in the plane normal to the principal loading direction, enabling 2D axi-symmetric finite element solutions to be used, as in reference [5], provided that all anisotropic materials are transverse isotropic about the loading direction. 

During the exploration of the initial design parameter space, when ball-park estimates of performance are all that is required, an approximate but fast method of analysis brings significant benefits by quickly narrowing down the extent of the parameter space that needs to be considered in detail. The analytical method described in reference [1], which is able to predict the response of a single stack of perfectly bonded multi-layers involving piezoelectric layers, will be the basis of an approximate model to predict the performance of the two more complex devices similar to those considered in reference [5]. Judicious approximations will be used to derive a simplified model of the devices capturing the essential features of device performance that will achieve a useful balance between the accuracy and the speed of analysis to help meet initial design requirements for devices that include three multi-layered stacks.

Multi-layered systems are very difficult to solve analytically because material property differences between layers lead to complex stress and displacement fields near the free edges. An analytical model of a multi-layered stack of cuboidal layers, including one or more piezoelectric layer(s) and associated electrodes, has already been developed [1] and verified by comparison of predictions with accurate FEA results. The analytical model, which assumes that the in-plane dimensions are much larger than the total thickness of the layers, attempts to describe the stress and displacement fields within interior regions of the laminate by assuming that the same in-plane and bending strains arise in all layers of the system. The consequence of this simplifying assumption is that the edges of the layered system must exhibit in-plane normal edge tractions and bending moments per unit area which are linear in each layer but discontinuous from layer to layer. The linear behaviour arises only if the multi-layered system is allowed to bend.

In Section 2 and Section 3, a compact approximate analytical model of a piezoelectric device is described and developed which is verified numerically in Section 4 and Section 5 by comparing some predictions of the analytical model with axi-symmetric FEA results based on special constrained boundary and interface conditions. Provided the compact model is used for equi-biaxial loading conditions, and provided anisotropic materials are transverse isotropic about the axis of symmetry of the device model, the compact model will predict solutions that are automatically axi-symmetric, enabling direct comparison of predictions with FEA solutions based on axisymmetric geometries. Conversely, solutions for axi-symmetric models can be used to predict the performance of corresponding multi-layered systems comprising cuboidal layers encountered in practice, provided equi-biaxial conditions prevail and anisotropic layers are transverse isotropic about the axis of symmetry.

**Figure 1:**
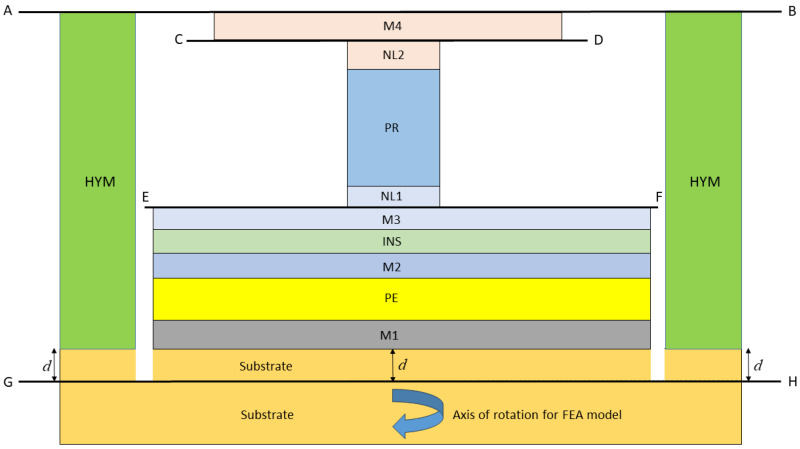
Schematic diagram of an axi-symmetric model of an RF switch (dimensions and some properties are given in Table 1).

**Figure 2:**
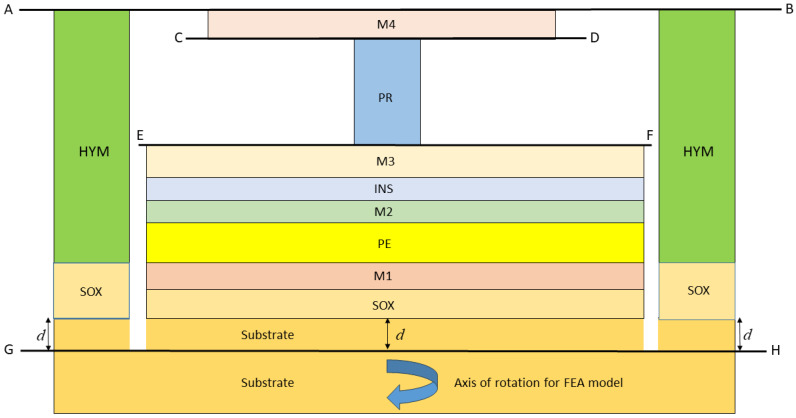
Schematic diagram of an axi-symmetric model of a VLSI device (dimensions and some properties are given in Table 2).

Two axi-symmetric FEA models are described in reference [5], where an RF switch (see Figure 1) is considered and a much smaller VLSI device (see Figure 2). Some modifications to the devices and the material properties have been made, leading to more challenging verification cases. Before considering the RF switch and VLSI devices in detail, it is useful to emphasise that the first principal objective is to verify that the compact model for the devices has been formulated and solved correctly and that the associated computer code is also correct. One very good way of achieving this is to undertake a finite element analysis of the devices so that the same geometry and material properties are used for both solution methods. In addition, as the compact model is an approximation, the boundary and interface assumptions needed to develop analytical solutions for the devices are replicated so far as is possible when developing FEA solutions. Very good agreement between predictions can then be expected, provided both solution techniques are free of errors, establishing a thorough verification of the modelling, both analytical and finite element analysis. For the verification stage of the modelling, the material properties have been represented as realistically as possible, given the anisotropic nature of some of the materials, e.g., the substrate, the PE material and the PR material.

Having verified the model by using FEA and constrained boundary and interface conditions, the second principal objective is to assess in Section 7 the practical utility of the compact model by comparing predictions of switching voltage described in Section 6 with FEA results where the constraints applied for verification have been relaxed so that FEA boundary and interface conditions are representative of those encountered in practice. It is emphasised that no attempt has been made to optimise the design of the devices considered, an issue that is beyond the scope to be covered herein.

The key advances beyond previously published work are the deployment of the model described in [1] to assess the ballpark performance of realistic nanoscale transistor devices and the comparison with FEA results to understand the limitations of the analytical approach and the root causes of the model’s limitations.

## 2. General Description and Material Properties for the Devices

Following the approach of reference [5], two types of devices will be considered herein. The first is an RF switch that is depicted schematically in Figure 1. The second is a much smaller VLSI device, as shown schematically in Figure 2. Table 1 and Table 2 provide details on the geometrical parameters and isotropic properties. The RF switch and VLSI device involve three stacks of layers where each stack can have a different number of layers, where the layers in each stack have the same internal/external radii (see Figure 1 and Figure 2).

The PE stack comprises six layers for the RF switch and seven layers for the VLSI device, all with the same radius. The lowest layer is part of the substrate with thickness *d*, which was selected to be large enough for the remainder of the substrate below the plane GH to be effectively in a stress-free state. The PR stack containing the PR layer, with three layers for the RF switch and a single layer for the VLSI device, has a much smaller radius than the PE layer so that the axial stresses generated are sufficient to operate a phase transition and associated resistance change within the PR layer. One layer (made of material M4) is immediately above the PR stack and its radius can differ from that of the PE layer. The third stack known as the yoke involves only two layers, one of which is made of substrate material. The inner radius of the yoke stack is clearly larger than the radius of the PE layer. The purpose of the yoke is to restrain the vertical movement of layer M4 in order to generate compressive axial stresses in the PR when the PE layer thickness increases as a result of an applied voltage across this layer.

### 2.1. Dimensions and Isotropic Properties for Device Models

The RF switch to be modelled is illustrated in Figure 1 and is a generalisation of the geometry assumed in reference [5], where a substrate of thickness *d* has been included in the model. The geometrical parameters and isotropic material properties are specified in Table 1.

For the RF switch shown in Figure 1, the difference between the outer and inner radii of the yoke stack is 400 nm and there is a 5 nm gap between the outer radius of the PE stack and the inner radius of the yoke stack. This means that the thickness of the yoke annulus is 400 nm. As the principal objective is to verify a compact model of the device, the properties are selected in this case so that neighbouring layers do not have the same properties.

The VLSI device to be modelled is illustrated in Figure 2 and it is noted, following the approach of reference [5], that the layers NL1 and NL2 used in the RF switch have been removed from the PR stack. In addition, a layer labelled SOX has been included in the PE stack. Part of the substrate with thickness *d* is, again, included in the model. The geometrical parameters and isotropic material properties are specified in Table 2.

For the VLSI device shown in Figure 2, the difference between the outer and inner radii of the yoke stack is 30 nm and there is a 10 nm gap between the outer radius of the PE stack and the inner radius of the yoke stack. This means that the thickness of the yoke annulus is 30 nm.

### 2.2. Anisotropic Properties

The contracted Voigt notation is used herein to describe the properties of anisotropic materials, although it is noted that the FEA software package Abaqus (release ID Abaqus 2021) uses a different contracted labelling system.

From Table 1 and Table 2, it is clear that the properties of the PR, PE and substrate are taken to be anisotropic and their values are specified and discussed in detail in the Supplementary Material (referring to references [6,7,8]). Anisotropic elastic properties are specified using either the elastic constant matrix $c_{ij}$ or the compliance matrix $s_{ij}$ which are inverses of one another. To maintain numerical precision, it is imperative that, if truncated values are given for one of these matrices, then truncation of the inverse values is avoided as this would introduce unwanted but avoidable errors into verification calculations. This is an important issue here because the analytical model for a single stack requires the compliance matrix $s_{ij}$ as input data while the FEA application Abaqus requires the elastic constant matrix $c_{ij}$ as input data.

A difficult issue arises when using property data in the literature for which both elastic moduli and compliance values are given for an anisotropic material where both data values are usually truncated. When comparing analytical model predictions with FEA results, it is clearly vital that a consistent set of property data is used. For the PE data [9] to be used in this case, the literature values of anisotropic properties are not consistent as the compliances and elastic moduli are not sufficiently accurate inverses of one another. This problem is investigated in detail in the Supplementary Material and a consistent set of anisotropic property data is specified and used for model verification.

Another issue concerns the need to use an axi-symmetric geometry when considering FEA models of the devices and the associated need to use anisotropic properties which are transverse isotropic about the axis of device symmetry. This is an issue, in particular, for the analytical models of stacks of cuboidal layers included in the devices, which are based on the compliance matrix $s_{ij}$ having equi-biaxial properties defined using Cartesian coordinates.

## 3. Development of an Equivalent 1D Model

**Figure 3:**
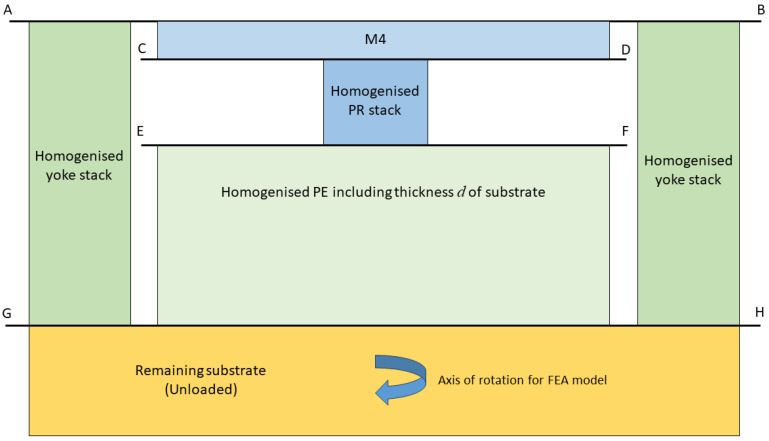
Schematic diagram of the axi-symmetric model of a device following homogenisation of the PE, PR, and yoke stacks.

The RF switch and VLSI device have a common electro-mechanical structure which will now be represented by a simplified 1D axi-symmetric compact model for such devices. The analytical approach to the development of a compact device model is first to homogenise using, in an axi-symmetric mode (i.e., equi-biaxial), the model of a single stack of cuboidal multi-layers based on the analysis given in reference [1]. This enables the calculation of the effective axial properties (mechanical, and piezoelectric when appropriate) of the PE and PR stacks for inclusion in the approximate compact model. The stacks of layers in the original device model can then be replaced by single layers with the effective properties of the layers in the device stacks that they replace, as shown schematically in Figure 3. The effective properties are then used to develop the following analysis that enables the determination of estimates of the axial stress state in all components of the device when a voltage is applied to the electrodes. The multi-layered model including piezo-electric layers, which was verified in reference [1], applies also to multi-layered linear elastic materials, so that it can also be used to homogenise the PR stack (for the RF switch) and the yoke stack. The effect of protective coatings can also be included in the model but they are not considered in this case.

It has already been stated in the Introduction that the analytical model is to be based on a special set of constrained boundary and interface conditions that were used in reference [1] when verifying the model of an isolated PE stack through comparison of predictions with those of FEA. Because the upper and lower surfaces of single stacks included in the two devices remain plane and parallel during deformation because of the applied constraint, it is possible to build a device of such stacks, as seen in Figure 3 where a PR stack is in series with a PE stack and in parallel with a two-layered yoke. The upper surface AB of the device is constrained to move uniformly in a vertical direction and its contact with both the top of the single layer M4 and the top layer of the yoke stack enables friction-free lateral slipping. Similarly, the lower surface CD of layer M4 and the upper layer of the PR stack are constrained to move uniformly in the vertical direction with friction-free lateral slipping. The same constraining conditions are applied on the surface EF between the lower surface of the PR stack and the upper layer of the PE stack. The lower surface of the PE stack and lower surface of the two-layer yoke are assumed to be constrained by a fixed base GH that allows friction-free sliding in a lateral direction. It is emphasised that the use of planar constraints combined with friction-free slipping are approximations that are required to enable the development of a device model with an analytical solution. The approximations enable the prediction of the uniform stress and strain distributions in each layer of the system when deformation is caused by the application of a voltage across the electrodes. In reality, the constrained conditions would be replaced by perfect bonding conditions between stacks leading to non-constrained complex non-uniform stress and deformation states in the layers of the stacks.

**Figure 4:**
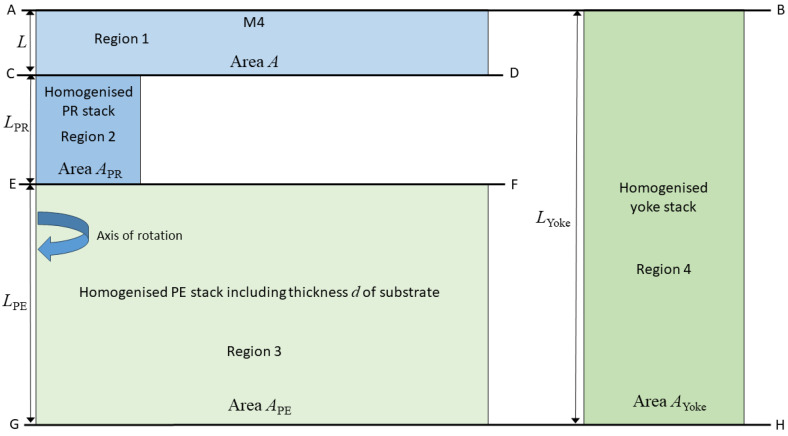
Schematic diagram of the simplified axi-symmetric 1D model where each region has unit thickness.

Having homogenised the layer stacks as indicated in Figure 3, the second step is to simplify the axi-symmetric model depicted in Figure 3 so that is replaced by Figure 4 which can be thought of as a schematic diagram of a 1D axi-symmetric parallel bar model, provided the areas *A*, $A_{\mathrm{PE}},$ $A_{\mathrm{PR}}$ and $A_{\mathrm{Yoke}}$ are calculated assuming the axi-symmetric geometry of Figure 3. The relevant geometrical parameters and four key elements of the devices labelled Regions 1–4 are illustrated in Figure 4. It is noted that Figure. 4 can also be thought of as representing a corresponding model of cuboidal elements as uniaxial loading is automatically equi-biaxial and provided that anisotropic layers are equi-biaxial with respect to the uniaxial loading direction. 

The element of the device representing the material M4 is labelled Region 1. It has height *L* and cross-sectional area *A*. The element that includes the PR is labelled Region 2 with height *L*_PR_ and cross-sectional area $A_{\mathrm{PR}}$ while the element that includes the PE is labelled Region 3 with height *L*_PE_ and cross-sectional area *A*_PE_. The fourth element labelled Region 4 represents the outer element of the axi-symmetric model shown in Figure 3 which is in fact an annulus with height $L_{\mathrm{Yoke}}=L+L_{\mathrm{PR}}+L_{\mathrm{PE}}$ and cross-sectional area *A*_Yoke_. The unloaded part of the silicon substrate has now been removed. The axial stress in each region is uniform and uniaxial as it is assumed that material on the upper and lower surfaces (AB and GH) and on the interfaces (CD and EF) can slide laterally without friction and vertically with uniform displacements.

The strain tensor is denoted by $S_{ij}$ and the corresponding stress tensor is denoted by $T_{ij}$. A superscript (I) is used to denote that the effective property refers to Region I where I  =  1,  2,  3,  4. For the analytical model, the stress–strain relations (which must be transverse isotropic about the axis of the devices) for the linear elastic homogenised material in Region I where I = 1, 2 and 4 are of the form (equi-biaxial):

(1) $$S_{11}=\;\;s_{11}^{\left(I\right)}T_{11}+s_{12}^{\left(I\right)}T_{22}+s_{13}^{\left(I\right)}T_{33},$$

(2) $$S_{22}=\;\;s_{12}^{\left(I\right)}T_{11}+s_{11}^{\left(I\right)}T_{22}+s_{13}^{\left(I\right)}T_{33},$$

(3) $$S_{33}=\;\;s_{13}^{\left(I\right)}T_{11}+s_{13}^{\left(I\right)}T_{22}+s_{33}^{\left(I\right)}T_{33}.$$

In Region 3, so that I  =  3, the constitutive relations for the homogenised PE material, with one piezoelectric layer, are given by (equi-biaxial)

(4) $$S_{11}=\;\;s_{11}^{E(3)}T_{11}+\;\;s_{12}^{E(3)}T_{22}+\;\;s_{13}^{E(3)}T_{33}+\;\;d_{31}^{\left(3\right)}E_{3},$$

(5) $$S_{22}=\;\;s_{12}^{E(3)}T_{11}+\;\;s_{11}^{E(3)}T_{22}+\;\;s_{13}^{E(3)}T_{33}+\;\;d_{31}^{\left(3\right)}E_{3},$$

(6) $$S_{33}=\;\;s_{13}^{E(3)}T_{11}+\;\;s_{13}^{E(3)}T_{22}+\;\;s_{33}^{E(3)}T_{33}+\;\;d_{33}^{\left(3\right)}E_{3},$$

(7) $$D_{3}=\varepsilon_{33}^{T(3)}E_{3}+\;\;d_{31}^{\left(3\right)}(T_{11}+T_{22})+d_{33}^{\left(3\right)}T_{33},$$

where $D_{3}$ and $E_{3}$ are respectively the through-thickness electric displacement and electric field components.

For the simplified model, the transverse stresses are assumed to be zero everywhere. The relations (1)–(7) then reduce to the form

Region 1:

(8) $$S_{11}=\;\;s_{13}^{\left(1\right)}T_{33},$$

(9) $$S_{22}=\;\;s_{13}^{\left(1\right)}T_{33},$$

(10) $$S_{33}=\;\;s_{33}^{\left(1\right)}T_{33},$$

Region 2:

(11) $$S_{11}=\;\;s_{13}^{\left(2\right)}T_{33},$$

(12) $$S_{22}=\;\;s_{13}^{\left(2\right)}T_{33},$$

(13) $$S_{33}=\;\;s_{33}^{\left(2\right)}T_{33},$$

Region 3:

(14) $$S_{11}=\;\;s_{13}^{E(3)}T_{33}+\;\;d_{31}^{\left(3\right)}E_{3},$$

(15) $$S_{22}=\;\;s_{13}^{E(3)}T_{33}+\;\;d_{31}^{\left(3\right)}E_{3},$$

(16) $$S_{33}=\;\;s_{33}^{E(3)}T_{33}+\;\;d_{33}^{\left(3\right)}E_{3},$$

(17) $$D_{3}=\varepsilon_{33}^{T(3)}E_{3}+d_{33}^{\left(3\right)}T_{33},$$

Region 4:

(18) $$S_{11}=\;\;s_{13}^{\left(4\right)}T_{33},$$

(19) $$S_{22}=\;\;s_{13}^{\left(4\right)}T_{33},$$

(20) $$S_{33}=\;\;s_{33}^{\left(4\right)}T_{33}.$$

The balance of total normal force at the interface between the PR and the M4 and PE materials requires that the non-zero stresses are such that

(21) $$AT_{33}^{\left(1\right)}=A_{\mathrm{PR}}T_{33}^{\left(2\right)}=A_{\mathrm{PE}}T_{33}^{\left(3\right)}.$$

As the system is unloaded vertically it follows that

(22) $$AT_{33}^{\left(1\right)}=A_{\mathrm{PR}}T_{33}^{\left(2\right)}=A_{\mathrm{PE}}T_{33}^{\left(3\right)}=-A_{\mathrm{Yoke}}T_{33}^{\left(4\right)}.$$

In addition, since the rigid top support can only move vertically and the fixed bottom support is prevented from moving vertically, it follows that

(23) $$LS_{33}^{\left(1\right)}+\;\;L_{\mathrm{PR}}S_{33}^{\left(2\right)}+\;\;L_{\mathrm{PE}}S_{33}^{\left(3\right)}=\;\;L_{\mathrm{Yoke}}S_{33}^{\left(4\right)}.$$

Upon using (10), (13), (16) and (20), it follows from (23) that

(24) $$Ls_{33}^{\left(1\right)}T_{33}^{\left(1\right)}+\;\;L_{\mathrm{PR}}s_{33}^{\left(2\right)}T_{33}^{\left(2\right)}+\;\;L_{\mathrm{PE}}\left(s_{33}^{E(3)}T_{33}^{\left(3\right)}+\;\;d_{33}^{\left(3\right)}E_{3}\right)=\;\;L_{\mathrm{Yoke}}s_{33}^{\left(4\right)}T_{33}^{\left(4\right)}.$$

Since $V=L_{\mathrm{PE}}E_{3}$ for infinitesimal deformations and upon using (22), the axial stresses $T_{33}^{\left(2\right)}$, $T_{33}^{\left(3\right)}$, and $T_{33}^{\left(4\right)}$ can be eliminated so that

(25) $$\left(\frac{L}{A}s_{33}^{\left(1\right)}+\;\;\frac{L_{\mathrm{PR}}}{A_{\mathrm{PR}}}s_{33}^{\left(2\right)}+\;\;\frac{L_{\mathrm{PE}}}{A_{\mathrm{PE}}}s_{33}^{E(3)}+\;\;\frac{L_{\mathrm{Yoke}}}{A_{\mathrm{Yoke}}}s_{33}^{\left(4\right)}\right)\;\;T_{33}^{\left(1\right)}=\;\;-\;\;\frac{V}{A}d_{33}^{\left(3\right)},$$

(26) $$T_{33}^{\left(2\right)}=\frac{A}{A_{\mathrm{PR}}}T_{33}^{\left(1\right)}\;,\;\;\;\;\;\;\;\;\;T_{33}^{\left(3\right)}=\frac{A}{A_{\mathrm{PE}}}T_{33}^{\left(1\right)}\;,\;\;\;\;\;\;\;\;\;T_{33}^{\left(4\right)}=-\frac{A}{A_{\mathrm{Yoke}}}T_{33}^{\left(1\right)}.$$

## 4. Finite Element Analysis

**Figure 5:**
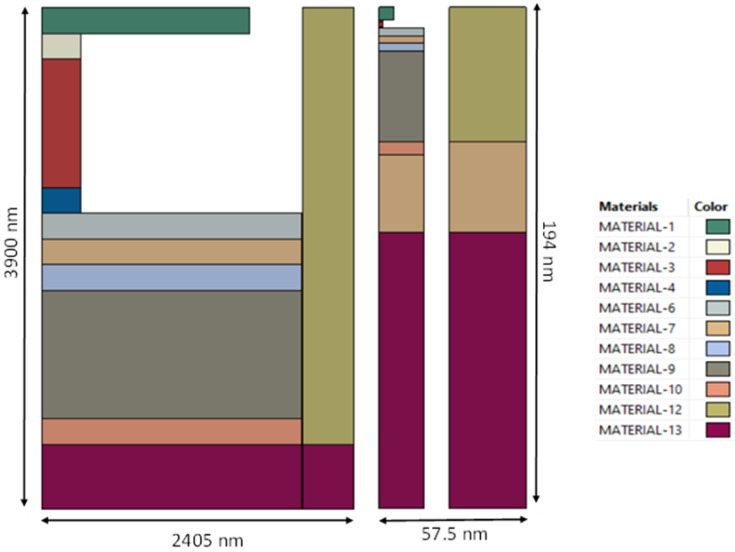
Axisymmetric model geometries of the RF switch and VLSI devices.

Finite element analysis (FEA) is a numerical technique for the solution of static stress problems that can be used to assess the accuracy of the analytical method of estimating the performance of the RF switch and VLSI device. A decision has to be made regarding the physical quantities that will be compared. As the devices are operated by using a voltage to generate compressive axial stresses in the PR, it is useful to focus on a comparison of the predictions of axial compressive stresses in layer M4, the PR stack, the PE stack and of the axial tensile stresses in the yoke stack. Because the device is completely stress free when the applied voltage is zero, it is sufficient to consider only a single non-zero applied voltage, which was selected as 1 V for all predictions of the compact and FEA models. The stresses generated upon applying any other voltage are all proportional to the value of that applied voltage. As already mentioned, the FEA predictions were made using the Abaqus FEA package, which helped to generate the schematic diagrams of the two axi-symmetric devices shown in Figure 5.

The devices are drawn to scale where the VLSI device has been enlarged so that it has the same height as the RF switch. The properties of the various isotropic materials and geometrical parameters are specified in Table 1 and Table 2, with transverse anisotropic properties being specified in the Supplementary Material. It is emphasised that there is a gap between the PE stack and yoke for the two cases considered, 5 nm for the RF switch and 10 nm for the VLSI device. The diagram shows that the PR stack of the RF switch has two ‘nails’ of equal depth of 200 nm, while these are absent for the VLSI device. The properties of the nails and the PR layer differ, as seen in Table 1.

### Constrained FEA Solutions for a Single Multi-Layered Stack

A special type of multi-layered problem that can be solved analytically assumes that bending during deformation on a single stack of layers is prevented by applying bending moments per unit area on the edges. This type of deformation can be replicated using FEA by applying a constraining set of boundary and interface conditions, as described in reference [1]. This is achieved by constraining the nodes on the top and bottom surfaces of the stack so that the surfaces remain plane during deformation in order that these plane surfaces can only stretch in the plane and move uniformly in the through-thickness direction. Constraint is also applied on the layer edges where all in-plane nodal surface displacements are required to have the same value, which ensures that the effective in-plane stresses are zero or equal to a prescribed value. Such constraints were applied in reference [1] using the Abaqus solver, and it was shown that the FEA results replicated analytical predictions, to within very small numerical errors. This procedure is regarded as the best method of verifying the solution and associated coding of the analytical model for a single stack of perfectly bonded layers containing one or more piezoelectric layers.

It will become clear that the use of constrained solutions to verify analysis methods, both analytical and FEA, provides a basis for the development of an approximate method of modelling the performance of micro-electronic devices of the types illustrated in Figure 1 and Figure 2 which exhibit two types of layer stacks. The first is the PE stack which contains one piezoelectric layer, while the other is the PR stack which can comprise three elastic materials or just one. It will now be shown that such an analysis is ideal as a basis for the development of a rapid method for assessing the performance characteristics of PET devices.

The axi-symmetric devices described in Section 2 have been modelled using the FEA system Abaqus [10]. The fully constrained FEA models replicate both the geometry and boundary conditions assumed by the analytical model as described in reference [1]. The analytical model constrains the various surfaces or interfaces shown in Figure 1 and Figure 2 as the planes AB, CD, EF, and GH so that they remain plane and parallel during deformation but allow for frictionless interface sliding. This constraint is imposed for the boundary and interface conditions that are applied to the FEA model when used for verification purposes. In order to replicate the nature of the deformation arising in the analytical model (i.e., uniform stress and strains within each layer of the device as bending is prevented), the external surfaces of the PR stack, the PE stack, and the yoke are constrained by the FEA model so that the radial displacement is uniform. The radial boundary conditions do not prescribe the actual distribution of the surface displacement as a single uniform value is applied that is calculated by the model so that the averaged radial traction acting on the external surface of each stack is zero.

In order to compare the FEA results with predictions using the compact model, values for the axial stresses along the axis of the PR, PE, and yoke stacks can be output in order to describe their distribution, which should be uniform in each layer when using constrained boundary and interface conditions.

## 5. Results of Model Verification

The RF switch illustrated in Figure 1 and the VLSI device shown in Figure 2 were modelled, for axi-symmetric conditions, using the FEA system Abaqus, in a way that should duplicate the results predicted by the compact model. For both models, the potential difference applied across the PE layer has a value of 1 V.

For the fully constrained FEA models, which replicate the compact model, all stresses and strains within each layer of the device are uniform, and this is confirmed by the FEA results, which are not shown. The fully constrained models are equi-biaxial. In order to compare the FEA results for this case with predictions using the compact model, values for the axial stresses in each region of the devices have been compared.

Table 3 and Table 4 show the axial stress values obtained for the RF switch and VLSI device respectively, together with the values of the percentage differences, which for the RF switch are close to 10^−4^, while for the VLSI device they are much smaller, with values close to 10^−5^. The decimal places that do not match in these tables are italicised. These results indicate that there is exceedingly good agreement between the FEA results and those provided by the compact model, and thus, it has been verified that both methodologies are consistent with one another and that the correct input data have been used.

## 6. Prediction of the Switching Voltages

Having verified the compact model convincingly by comparing predictions of axial stresses with the results of finite element analysis using special boundary and interface conditions designed to replicate the results of the compact model, an attempt is now made to assess the performance of the compact model when compared to the FEA results by focusing on the switching voltage (as in reference [5]).

### 6.1. Calculation of the Hydrostatic Pressure in the PR

The calculation of the switching voltage requires knowledge of the pressure state in the PR layer. For the VLSI device, the PR stack has one layer made entirely of the PR material. The approximate analytical model predicts that the pressure will then be uniform within the PR and is easily calculated. However, for the RF switch, two nails are included, as shown in Figure 1, and a more involved stress analysis is required that attempts to account for the presence of the nails. An approximate analysis will now be described that follows the same approach as used for the analytical model applied to the PE and yoke stacks and is consistent with the FEA analysis when using constrained boundary and interface conditions.

The following analysis applies to two perfectly bonded layers representing the three layers comprising the PR stack. The upper and lower layers are identical while the central layer is made of PR material. The upper and lower layers can be combined into a single layer with the combined thickness. The system is subject to a uniform temperature change Δ*T*, a uniform axial stress σ, and a uniform transverse stress $\sigma_{T}=0$ applied on the external surface of the PR stack. A set of Cartesian coordinates $\left(x_{1},\;x_{2},\;x_{3}\right)$ will be used.

The PR is regarded as a transverse isotropic solid so that the stress–strain-temperature relations are of the form:

(27) $$\begin{array}{l} \varepsilon_{11}^{\mathrm{PR}}=s_{11}^{\mathrm{PR}}\sigma_{11}^{\mathrm{PR}}+\;s_{12}^{\mathrm{PR}}\sigma_{22}^{\mathrm{PR}}+\;s_{13}^{\mathrm{PR}}\sigma_{33}^{\mathrm{PR}}+\;\alpha_{T}^{\mathrm{PR}}\Delta T,\\ \varepsilon_{22}^{\mathrm{PR}}=s_{12}^{\mathrm{PR}}\sigma_{11}^{\mathrm{PR}}+\;s_{11}^{\mathrm{PR}}\sigma_{22}^{\mathrm{PR}}+\;s_{13}^{\mathrm{PR}}\sigma_{33}^{\mathrm{PR}}+\;\alpha_{T}^{\mathrm{PR}}\Delta T,\\ \varepsilon_{33}^{\mathrm{PR}}=\;s_{13}^{\mathrm{PR}}\sigma_{11}^{\mathrm{PR}}+\;s_{13}^{\mathrm{PR}}\sigma_{22}^{\mathrm{PR}}+\;s_{33}^{\mathrm{PR}}\sigma_{33}^{\mathrm{PR}}+\;\alpha_{A}^{\mathrm{PR}}\Delta T\;. \end{array}$$

Nails NL1 and NL2 are also regarded as a transverse isotropic solid made of the same material so that

(28) $$\begin{array}{l} \varepsilon_{11}^{\mathrm{NL}}=s_{11}^{\mathrm{NL}}\sigma_{11}^{\mathrm{NL}}+\;s_{12}^{\mathrm{NL}}\sigma_{22}^{\mathrm{NL}}+\;s_{13}^{\mathrm{NL}}\sigma_{33}^{\mathrm{NL}}+\;\alpha_{T}^{\mathrm{NL}}\Delta T,\\ \varepsilon_{22}^{\mathrm{NL}}=s_{12}^{\mathrm{NL}}\sigma_{11}^{\mathrm{NL}}+\;s_{11}^{\mathrm{NL}}\sigma_{22}^{\mathrm{NL}}+\;s_{13}^{\mathrm{NL}}\sigma_{33}^{\mathrm{NL}}+\;\alpha_{T}^{\mathrm{NL}}\Delta T,\\ \varepsilon_{33}^{\mathrm{NL}}=s_{13}^{\mathrm{NL}}\sigma_{11}^{\mathrm{NL}}+\;s_{13}^{\mathrm{NL}}\sigma_{22}^{\mathrm{NL}}+\;s_{33}^{\mathrm{NL}}\sigma_{33}^{\mathrm{NL}}+\;\alpha_{A}^{\mathrm{NL}}\Delta T\;. \end{array}$$

Throughout the nails and PR material, the axial stress is uniform and equal to the axial stress predicted by the analytical multi-layer model so that

(29) $$\sigma_{33}^{\mathrm{PR}}=\;\;\sigma ,\;\;\;\;\;\;\;\;\;\;\;\;\;\;\;\;\;\;\;\;\;\;\;\;\sigma_{33}^{\mathrm{NL}}=\;\;\sigma \;,$$

where σ is the known value of the axial stress predicted for the PR stack when using the analysis given in Section 3. An approximate solution to the PR stack problem is now sought of the following form:

(30) $$\begin{array}{cc} u_{1}^{\mathrm{PR}}=A\;x_{1},\;u_{2}^{\mathrm{PR}}=A\;x_{2}\;, & u_{1}^{\mathrm{NL}}=A\;x_{1},\;\;u_{2}^{\mathrm{NL}}=Ax_{2}\;,\\ u_{3}^{\mathrm{PR}}\equiv \varepsilon_{\mathrm{PR}}x_{3}\;, & u_{3}^{\mathrm{NL}}\equiv \varepsilon_{\mathrm{NL}}x_{3}\;, \end{array}$$

where $\varepsilon_{\mathrm{PR}}$ is the axial strain in the PR and $\varepsilon_{\mathrm{NL}}$ is the axial strain in the nails where *A* is a constant that is to be determined. On differentiating the displacement field it follows that

(31) $$\begin{array}{l} \varepsilon_{11}^{\mathrm{PR}}=\frac{\partial u_{1}^{\mathrm{PR}}}{\partial x_{1}}=A,\varepsilon_{22}^{\mathrm{PR}}=\frac{\partial u_{2}^{\mathrm{PR}}}{\partial x_{2}}=A,\varepsilon_{33}^{\mathrm{PR}}=\varepsilon_{\mathrm{PR}},\;\\ \varepsilon_{11}^{\mathrm{NL}}=\frac{\partial u_{1}^{\mathrm{NL}}}{\partial x_{1}}=A,\varepsilon_{22}^{\mathrm{NL}}=\frac{\partial u_{2}^{\mathrm{NL}}}{\partial x_{2}}=A,\varepsilon_{33}^{\mathrm{NL}}=\varepsilon_{\mathrm{NL}}\;. \end{array}$$

It is easily shown from (27) and (28) that

(32) $$\sigma_{22}^{\mathrm{PR}}=\;\sigma_{11}^{\mathrm{PR}},\;\;\;\;\;\;\;\;\;\;\;\;\sigma_{22}^{\mathrm{NL}}=\;\sigma_{11}^{\mathrm{NL}},$$

and that these stress–strain relations may then be reduced to the following four independent equations:

(33) $$\begin{array}{l} A\;=\left(s_{11}^{\mathrm{PR}}+\;s_{12}^{\mathrm{PR}}\right)\sigma_{11}^{\mathrm{PR}}+\;s_{13}^{\mathrm{PR}}\sigma +\;\alpha_{T}^{\mathrm{PR}}\Delta T,\\ A\;=\left(s_{11}^{\mathrm{NL}}+\;s_{12}^{\mathrm{NL}}\right)\sigma_{11}^{\mathrm{NL}}+\;s_{13}^{\mathrm{NL}}\sigma +\;\alpha_{T}^{\mathrm{NL}}\Delta T,\\ \varepsilon_{\mathrm{PR}}=\;2s_{13}^{\mathrm{PR}}\sigma_{11}^{\mathrm{PR}}+\;s_{33}^{\mathrm{PR}}\sigma +\;\alpha_{A}^{\mathrm{PR}}\Delta T\;,\\ \varepsilon_{\mathrm{NL}}=2s_{13}^{\mathrm{NL}}\sigma_{11}^{\mathrm{NL}}+\;s_{33}^{\mathrm{NL}}\sigma +\;\alpha_{A}^{\mathrm{NL}}\Delta T\;. \end{array}$$

A subtraction of the first two equations then leads to

(34) $$\begin{array}{l} \left(s_{11}^{\mathrm{PR}}+\;s_{12}^{\mathrm{PR}}\right)\sigma_{11}^{\mathrm{PR}}+\;s_{13}^{\mathrm{PR}}\sigma +\;\alpha_{T}^{\mathrm{PR}}\Delta T=\;\;\left(s_{11}^{\mathrm{NL}}+\;s_{12}^{\mathrm{NL}}\right)\sigma_{11}^{\mathrm{NL}}+\;s_{13}^{\mathrm{NL}}\sigma +\;\alpha_{T}^{\mathrm{NL}}\Delta T,\\ \;\;\;\;\;\;\;\;\;\;\;\;\;\;\;\;\;\;\varepsilon_{\mathrm{PR}}=\;2s_{13}^{\mathrm{PR}}\sigma_{11}^{\mathrm{PR}}+\;s_{33}^{\mathrm{PR}}\sigma +\;\alpha_{A}^{\mathrm{PR}}\Delta T\;,\\ \;\;\;\;\;\;\;\;\;\;\;\;\;\;\;\;\;\;\varepsilon_{\mathrm{NL}}=2s_{13}^{\mathrm{NL}}\sigma_{11}^{\mathrm{NL}}+\;s_{33}^{\mathrm{NL}}\sigma +\;\alpha_{A}^{\mathrm{NL}}\Delta T\;. \end{array}$$

There are, however, four unknowns which are $\sigma_{11}^{\mathrm{PR}},\;\;\;\sigma_{11}^{\mathrm{NL}},\;\;\varepsilon_{\mathrm{PR}}$, and $\varepsilon_{\mathrm{NL}}$. An additional relation is therefore needed, which is selected to ensure that the net transverse forces applied in the $x_{1}$ and $x_{2}$ directions to the PR stack are both zero. It is clear that

(35) $$t_{\mathrm{PR}}\sigma_{11}^{\mathrm{PR}}+\;\;2t_{\mathrm{NL}}\sigma_{11}^{\mathrm{NL}}=\;\;0,$$

where $t_{\mathrm{PR}}$ and $t_{\mathrm{NL}}$ are the thicknesses of the PR and each nail, respectively. The factor 2 appears in (35) because there are two identical nails in the PR stack, above and below the PR material. It then follows that the simultaneous linear equations specified by (34) and (35) may be solved so that

(36) $$\begin{array}{l} \left[\left(s_{11}^{\mathrm{PR}}+\;s_{12}^{\mathrm{PR}}\right)\;\;+\;\;\frac{t_{\mathrm{PR}}}{2t_{\mathrm{NL}}}\left(s_{11}^{\mathrm{NL}}+\;s_{12}^{\mathrm{NL}}\right)\right]\;\;\sigma_{11}^{\mathrm{PR}}=\;\;\left(s_{13}^{\mathrm{NL}}-\;s_{13}^{\mathrm{PR}}\right)\sigma +\;\left(\alpha_{T}^{\mathrm{NL}}-\;\alpha_{T}^{\mathrm{PR}}\right)\Delta T,\\ \;\;\;\;\;\;\;\;\;\;\;\;\;\;\;\;\;\;\;\;\varepsilon_{\mathrm{PR}}=\;2s_{13}^{\mathrm{PR}}\sigma_{11}^{\mathrm{PR}}+\;s_{33}^{\mathrm{PR}}\sigma +\;\alpha_{A}^{\mathrm{PR}}\Delta T\;,\\ \;\;\;\;\;\;\;\;\;\;\;\;\;\;\;\;\;\;\;\;\varepsilon_{\mathrm{NL}}=-\frac{t_{\mathrm{PR}}}{t_{\mathrm{NL}}}s_{13}^{\mathrm{NL}}\sigma_{11}^{\mathrm{PR}}+\;s_{33}^{\mathrm{NL}}\sigma +\;\alpha_{A}^{\mathrm{NL}}\Delta T\;. \end{array}$$

From (29) and (32), it follows that $\sigma_{22}^{\mathrm{PR}}=\;\sigma_{11}^{\mathrm{PR}},\;\;\;\;\sigma_{22}^{\mathrm{PR}}=\sigma ,$ so that the stresses in the PR element of the PR stack are thus determined. The hydrostatic pressure in the PR is then given by

(37) $$p_{\mathrm{PR}}=-\frac{1}{3}\left(\sigma_{11}^{\mathrm{PR}}+\;\;\sigma_{22}^{\mathrm{PR}}+\;\;\sigma_{33}^{\mathrm{PR}}\right)=-\frac{1}{3}\left(2\sigma_{11}^{\mathrm{PR}}+\;\;\sigma \right).$$

### 6.2. Estimation of the Switching Voltage

Both an RF switch and a VLSI device will be considered, using the same material properties and geometries that were used for model verification. Following the approach described in reference [5], including its Supplementary Material, it is clear from reference [11] (inset of Figure 1 therein), that the resistivity of the PR material made of SmSe decreases dramatically if sufficient pressure is applied. When the applied voltage V=0 such that the pressure in the PR is zero and the resistivity $\rho_{0}$ of the PR has the value of 3.5 Ω.cm. Upon applying a pressure of 45 kbar (4.5 GPa) to the PR, the resistivity plummets to the value of 10^−4^ Ω.cm, enabling an electric current to pass. The log_10_—linear plot of the resistivity of SmSe as a function of the pressure is linear for the pressure range 0–20 kbar (0–2 GPa). Assuming that the resistivity vs. pressure data are sufficiently well represented by such a linear relation for all pressures, it follows that (c.f. reference [5], text following Equation (1))

(38) $$\frac{\rho }{\rho_{0}}\;\;≅\;\;\exp\left(-\;B_{h}p_{\mathrm{PR}}\right),$$

where the value of the constant $B_{h}$ with the dimension (stress)^−1^ is determined from the slope of the log_10_—linear plot of the resistivity versus pressure curve given in reference [11]. The pressure in the PR is calculated using

(39) $$p_{\mathrm{PR}}=-\frac{1}{3}\mathrm{trace}\left(T_{\mathrm{PR}}\right),$$

where, for the FEA model, $T_{\mathrm{PR}}$ is the average of the stress tensor over the mid-horizontal surface of the PR, which depends implicitly on the applied voltage *V*. The approach assumes that the current density is constant in the PR, as is certainly the case when using (39) in conjunction with the analytical model. In reference [5], it is stated that the value of the constant $B_{h}=\;\;5.25\;\;{\mathrm{GPa}}^{-1}$ is taken from the slope of the resistivity-versus-pressure curve in references [11,12]. When thermal residual stresses and lattice mismatch stresses are zero, the pressure in the PR is simply proportional to the voltage *V*. It then follows from (38) that

(40) $$\frac{\rho (V)}{\rho_{0}}\;\;≅\;\;\exp\left(-B_{h}\;{\hat{p}}_{\mathrm{PR}}\;V\right),$$

where ${\hat{p}}_{\mathrm{PR}}$ is the pressure in the PR when a unit voltage is applied whose value is readily obtained from both the analytical and FEA models.

The ‘On’ voltage $V_{\mathrm{On}}$, enabling the passage of a current through the PR, is selected so that $\rho (V_{\mathrm{On}})/\rho_{0}=10^{-4}$, in which case it follows from (40) that the switching voltage is predicted to have the value

(41) $$V_{\mathrm{On}}=\;\;\frac{\ln\;(10^{4})}{B_{h}{\hat{p}}_{\mathrm{PR}}}$$

## 7. Assessment of Device Performance Using the Compact Model and FEA Methods

Based on the analysis described in Section 6, it is now possible to use the compact model of a device, schematically represented in Figure 4, to estimate the switching voltages for both the RF switch and the VLSI device. When using the compact model to predict device performance, the constraints applied to boundary and interface conditions must continue to be used as they are the fundamental basis for the development of the approximate analytical model. However, when using finite element methods to predict device performance, physically realistic boundary conditions must be applied where all interfaces are perfectly bonded, where bending of individual layers is allowed, and where free surfaces require the application locally of zero normal traction boundary conditions. The relaxation of the constraints applied to finite element predictions will lead to differing results and to larger differences between the predictions of FEA and the approximate compact model of the devices. These differences are now investigated and will include a consideration of the effect of the thickness of the PE layer. For the RF switch, PE thicknesses of 500 nm and 1000 nm (the model verification value) will be considered. The substrate thickness is selected so that the total height of the RF switch model is the same value of 3900 nm. For the VLSI device, PE thicknesses of 35 nm (the verification value) and 70 nm are considered, with the substrate thickness being selected so that the total height of the VLSI model is 194 nm. The predictions of the compact model and the corresponding FEA results based on realistic boundary and interface conditions are shown in Table 5.

It is clear, when considering the VLSI device for two PE thicknesses, that the switching voltage is grossly over-estimated by the approximate compact model when compared with the FEA predictions that use realistic boundary and interface conditions. This is an unacceptable situation if using the compact model to screen initial device designs. On the other hand, for the RF switch, the compact model leads to percentage errors that are acceptable for screening purposes. When the PE thickness is 500 nm, the switching voltage is under-estimated and the magnitude of the error is less than 4%. When the PE thickness is 1000 nm, the switching voltage is over-estimated and the magnitude of the error is about 38%.

**Figure 6:**
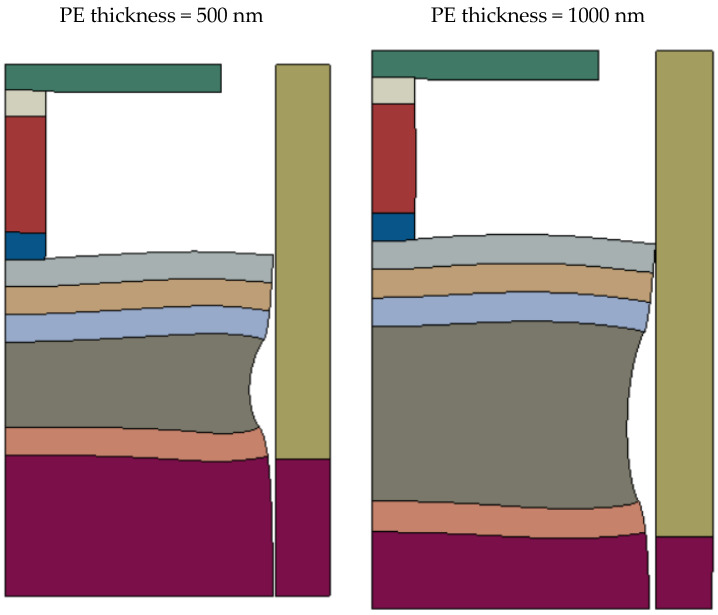
Deformation of the axi-symmetic model of the RF switch (magnified 500 times) predicted by the FEA model using realistic boundary and interface conditions.

An attempt is now made to explain the wide range of performance of the approximate compact model of the devices when compared to accurate FEA predictions. To gain some insight into this behaviour in the case of the RF switch, it is useful to examine the deformation predicted by FEA when using the realistic unrestrained boundary and interface conditions applied to the axi-symmetric model. The deformation is illustrated in Figure 6 where all displacements have been magnified by a factor of 500 in order to exaggerate the displacement values.

Figure 6 shows two types of behaviour that the approximate compact model is designed to avoid. The first concerns the localised bending of layers which can be seen in all layers having the same radius as the PE. The second type of behaviour concerns the deformation of the external surface of the PE stack. When the 1 V potential difference is applied, the PE layer thickens in order to generate compressive stress in the PR stack, leading to a Poisson radial contraction, which is clearly seen. In sharp contrast, the approximate compact model solution does not exhibit any layer bending, and all layers in both the PR and PE stacks have the same uniform Poisson contraction as transverse frictionless slipping is allowed on the interfaces between the PR, PE, and yoke stacks.

To gain some insight into this behaviour in the case of the VLSI device, an unsuccessful attempt was made to generate results similar to those for the RF switch shown in Figure 6. The ratio of the PR radius to the radius of the PE stack is about half that for the RF switch, and this leads to an apparent overlapping material when the deformation is magnified and to diagrams that appear anomalous.

The schematic diagrams of the two devices shown in Figure 5 indicate that there is a significant difference between the radii of the PE and PR stacks. For the RF switch, the ratio of the radius of the PR stack to that of the PE stack is 0.15, while for the VLSI device, the ratio is 0.086 which is approximately half the RF switch value. For the RF switch, it is remarkable that the percentage differences shown in Table 5 are as low as estimated, indicating that, for many multi-layered devices of similar construction, the compact model should be a reliable screening tool that can help to reduce the parameter design space to a limited set of parameter values where FEA can then be focused. This is clearly not the case for the VLSI device where the percentage errors are too large. The geometry of the VLSI device is such that the transverse dimensions of the PR element are much smaller than the transverse dimensions of neighbouring layers. This is a challenging situation for the validity of the compact model as the geometry in Figure 5 suggests that the small PR is acting more as a point force rather than a distributed load. Indeed, the PR appears more like an indentation probe into a flat surface rather than being a perfectly bonded neighbouring layer. It is, perhaps, not surprising that the VLSI device leads to the rather large percentage errors in Table 5 when using the compact model, which is not designed to deal with indentation scenarios.

The reason for the significant discrepancies between the FEA and compact model predictions arises principally because the verification results use freely slipping interface conditions between the device elements where bending of the layers is prevented. The corresponding FEA results assume perfectly bonded conditions that prevent interface slipping but allow for bending of the layers. Also, the compact model is not able to model edge effects that arise because of property differences between individual perfectly bonded layers in the PE and PR stacks. The extent of edge effects is usually determined by the thicknesses of the layers. Perfectly bonded FEA solutions do lead to such edge effects, which are characterised by complex localised stress and deformation non-uniformities that are not present when using the compact model which assumes freely-slipping boundaries between device elements without bending.

## 8. Conclusions

The principal objectives have been the development of an approximate compact model to predict the performance of micro-electronic devices and the comparison of predictions with accurate results obtained using FEA. Two types of FEA are performed using different types of boundary and interface conditions. The first type introduces conditions that are designed to replicate the behaviour of the compact model. Conditions enabling transverse slipping but without bending are imposed on selected interfaces between device components, while the radial displacements on each of the external surfaces of the PR, PE and yoke stacks are constrained to have specific values that are consistent with a zero effective normal traction when tractions are averaged over the surface where the conditions are imposed. For these types of boundary and interface conditions, the compact model predictions and FEA results should be identical, apart from numerical errors. This is an important issue as the input of property data (especially electro-thermo-elastic constants) and geometrical parameters is very complex and consistency between parameters used for both the compact model and FEA needs to be readily demonstrated. The results in Table 3 and Table 4 indicate that consistency has been achieved when the approach has been applied to an RF switch with a PE thickness of 1000 nm and to a VLSI device with a PE thickness of 70 nm. This verifies that consistent input data (both geometry and properties) have been applied to the compact and FEA models and that the compact models for the two devices have been formulated correctly.

The second type of boundary and interface condition is designed to replicate the actual behaviour of the devices in practice. The boundary and interface constraints applied for the verification procedure are relaxed so that bending of layers can occur, but with perfect interface bonding. The external surfaces of the PR, PE and yoke stacks are unconstrained so that the radial tractions are zero at all points of these surfaces. As to be expected, the approximate compact model predictions no longer agree with the FEA results, as seen from the results shown in Table 5. For this unconstrained case, the resulting deformation is very complex, involving both bending effects that are not modelled by the compact model, and edge effects where stress transfer between adjacent layers occurs near free surfaces due to the property mismatches between neighbouring layers. The results given in Table 5 for the RF switch show surprisingly good agreement between the predictions of the compact and FEA results, especially for the case when the PE thickness is 500 nm implying that the analytical model should help to reduce the parameter design space. However, for the VLSI device, the results in Table 5 indicate that the compact model leads to much larger errors. This is not unexpected as the PR is acting more like a point force than a neighbouring perfectly bonded layer with distributed surface tractions. For such systems, the compact model is unlikely to be able to reliably reduce the parameter design space, implying that accurate FEA will then need to be used over a much wider range of parameter values.

Results presented, based on the use of constrained boundary and interface conditions, regarding verification of both compact models and FEA have been established for relatively simple microelectronic devices involving multi-layered components. It is emphasised that the same methods can be used to verify models where various multi-layered components are integrated to form more complex devices.

The model also offers opportunities to rapidly and efficiently explore effects that are well known to affect device performance. One example is the presence of dopants which affect the material properties of a given layer. The compact model that is the basis of this work allows the user to assess rapidly how variation in these properties affects the switching voltage or the axial stresses in the layer stacks. This allows the model user to understand whether dopants or other impurities will have a significant effect on device performance, enabling the specification of material purity requirements at an early stage of device development.

## Figures and Tables

**Table 1:** Dimensions and properties of materials (isotropic only) used in the RF switch.

Layer Material	Outer Radius (nm)	Height (nm)	Young’s Modulus (GPa)	Poisson’s Ratio
M4	1600	200	200	0.25
NL2	300	200	530	0.3
PR	300	1000	Anisotropic	Anisotropic
NL1	300	200	530	0.3
M3	2000	200	400	0.35
INS	2000	200	200	0.25
M2	2000	200	530	0.3
PE	2000	1000	Anisotropic	Anisotropic
M1	2000	200	170	0.38
Substrate	2000	500 (=*d*)	Anisotropic	Anisotropic
HYM	2405	3400	530	0.3
Yoke substrate	2405	500 (=*d*)	Anisotropic	Anisotropic

**Table 2:** Dimensions and properties of materials (isotropic only) used in the VLSI device.

Layer Material	Outer Radius (nm)	Height (nm)	Young’s Modulus (GPa)	Poisson’s Ratio
M4 (Ir)	6	5	530	0.25
PR (SmSe)	1.5	3	Anisotropic	Anisotropic
M3 (Ir)	17.5	3	530	0.25
INS (SiN)	17.5	3	200	0.25
M2 (Pt)	17.5	3	170	0.38
PE (PMN-Pt)	17.5	35	Anisotropic	Anisotropic
M1 (Pt)	17.5	5	170	0.38
SOX (SiN)	17.5	30	200	0.25
Substrate (Si [110])	17.5	107 (=*d*)	Anisotropic	Anisotropic
Yoke HYM (SiN)	57.5	52	200	0.25
Yoke SOX (SiN)	57.5	35	200	0.25
Yoke substrate (Si [110])	57.5	107 (=*d*)	Anisotropic	Anisotropic

**Table 3:** Comparison of axial stresses for the RF switch (digits in italics highlight differences between the compact model and FEA predictions).

Region	Compact Model (GPa)	FEA (GPa)	Magnitude of % Difference
M4 (top)	−1.20785*17* × 10^−3^	−1.20785*297* × 10^−3^	1.05 × 10^−4^
PR stack	−3.4356*671* × 10^−2^	−3.4356*7021* × 10^−2^	0.905 × 10^−4^
PE stack	−7.73025*09* × 10^−4^	−7.73025*968* × 10^−4^	1.14 × 10^−4^
Yoke stack	1.75289*14* × 10^−3^	1.75289*321* × 10^−3^	1.03 × 10^−4^

**Table 4:** Comparison of axial stresses for the VLSI device (digits in italics highlight differences between the compact model and FEA predictions).

Region	Compact Model (GPa)	FEA (GPa)	Magnitude of % Difference
M4 (top)	−0.5457555*5*	−0.5457555*1*	8.06 × 10^−6^
PR stack	−8.732088*9*	−8.732088*1*	9.28 × 10^−6^
PE stack	−0.0641541*22*	−0.0641541*33*	1.75 × 10^−5^
Yoke stack	0.00770478*43*	0.00770478*37*	7.79 × 10^−6^

**Table 5:** Switching voltages for the RF switch and VLSI device.

Device (PE Layer Thickness)	Compact Model	FEA Model	Magnitude of % Difference
RF switch (500 nm)	163.02204 V	169.4606 V	3.8
RF switch (1000 nm)	146.02465 V	105.5251 V	38.38
VLSI device (35 nm)	0.602725 V	0.167211 V	260
VLSI device (70 nm)	0.609608 V	0.201502 V	202

## Data Availability

The original contributions presented in the study are included in the article/Supplementary Material, further inquiries can be directed to the corresponding author.

## Supplementary Materials

The following supporting information can be downloaded at: https://www.mdpi.com/article/10.3390/mi16020114/s1.
