# Supplementary material for: A Compact Device Model for a Piezoelectric Nano-Transistor
Source: Micromachines (Basel). 2025 Jan 21;16(2):114. doi: 10.3390/mi16020114 (PMC11857766; doi:10.3390/mi16020114)
Supplement: Supplementary file 1 [file micromachines-16-00114-s001.zip › micromachines-3306591-supplementary.pdf]

Supplementary Information: A compact device model for a piezoelectric nano-transistor

Anisotropic properties for model verification

Given that many materials used in MEMS devices are anisotropic, for example, the substrate, PR, and PE, it is essential that the analytical model be verified for realistic conditions involving such anisotropic materials. It is noted that the analytical model does not make use of elastic shear properties, but such properties are required by finite element analysis. The elastic constants data used in simulations will be enclosed in a box, where both c_{ij}^E and s_{ij}^E values are given, which are exact inverses of each other to within numerical errors.

S1. Properties of silicon

The substrate is treated as an elastic solid with a cubic symmetry [1,0,0] using values typical of silicon (diamond cubic structure). The elastic constants (using Voigt notation) with a cubic symmetry (see for example [6, 7])

$$\begin{aligned} c_{11} &= c_{22} = c_{33} = 1.657 \text{ x } 10^{11} \text{ Pa} , \\ c_{12} &= c_{13} = c_{23} = 0.639 \text{ x } 10^{11} \text{ Pa} , \\ c_{44} &= c_{55} = c_{66} = 0.796 \text{ x } 10^{11} \text{ Pa} . \end{aligned} \tag{S1}$$

These values are equivalent to the following three independent elastic constants:

$$\text{Young's modulus} = 130.13 \text{ GPa}, \;\; \text{Poisson's ratio} = 0.278, \;\; \text{Shear modulus} = 79.6 \text{ GPa}.$$

Upon inverting the moduli matrix c_{ij}, the values of the components of the compliance matrix (from code) are given by

$$\begin{aligned} s_{11} &= s_{22} = s_{33} = \;\; 0.7684506816 \text{ x } 10^{-11} \text{ (Pa}^{-1}) , \\ s_{12} &= s_{13} = s_{23} = -\, 0.2138675895 \text{ x } 10^{-11} \text{ (Pa}^{-1}) , \\ s_{44} &= s_{55} = s_{66} = \;\; 1.256281407 \text{ x } 10^{-11} \text{ (Pa}^{-1}) . \end{aligned} \tag{S2}$$

For silicon [1,1,0], a common orientation for MEMS devices, the elastic compliances are denoted by s'_{ij} where the compliance matrix $\boldsymbol{s}$ generated by (S2) has been rotated by an angle of $\pi / 4$ about the x_3 axis. The components of the transformed compliance matrix s'_{ij} then have the values

$$\begin{aligned} & s'_{11} = s'_{22} = \tfrac{1}{2} s_{11} + \tfrac{1}{2} s_{12} + \tfrac{1}{4} s_{66}, \qquad s'_{33} = s_{33} , \\ & s'_{12} = \tfrac{1}{2} s_{11} + \tfrac{1}{2} s_{12} - \tfrac{1}{4} s_{66}, \quad s'_{13} = s_{13} , \quad s'_{23} = s_{13} , \\ & s'_{16} = 0, \qquad s'_{26} = 0, \qquad s'_{36} = 0, \quad s'_{45} = 0 , \\ & s'_{44} = s_{44} , \qquad s'_{55} = s_{44} , \qquad s'_{66} = 2(s_{11} - s_{12}). \end{aligned} \tag{S3}$$

Upon substituting the values given by (S2), without any rounding, into (S3), the following non-zero values of s'_{ij} result

$$
\begin{aligned}
&s'_{11} = s'_{22} = 0.5913618978 \text{ x } 10^{-11}, && s'_{33} = 0.7684506816 \text{ x } 10^{-11} && (\text{Pa}^{-1}), \\
&s'_{12} = -\ 0.0367788057 \text{ x } 10^{-11}, && s'_{13} = s'_{23} = -0.2138675895 \text{ x } 10^{-11} && (\text{Pa}^{-1}), \\
&s'_{44} = s'_{55} = 1.256281407 \text{ x } 10^{-11}, && s'_{66} = 1.964636542 \text{ x } 10^{-11} && (\text{Pa}^{-1}).
\end{aligned}
\tag{S4}
$$

The values lead to the following Young's moduli, Poisson's ratios, and shear moduli:

$$
\begin{aligned}
&E_{11} = E_{22} = 169.1011889 \text{ GPa}, && E_{33} = 130.1319686 \text{ GPa}, \\
&\nu_{12} = 0.06219339774, && \nu_{13} = \nu_{23} = 0.3616526366, \\
&\mu_{12} = 50.9 \text{ GPa}, && \mu_{13} = \mu_{23} = 79.6 \text{ GPa}.
\end{aligned}
\tag{S5}
$$

Upon inverting the matrix s'_{ij} defined by (S4), the following non-zero values of c'_{ij} result:

$$
\begin{aligned}
&c'_{11} = c'_{22} = 1.944 \text{ x}10^{11}, && c'_{33} = 1.657 \text{ x}10^{11} && (\text{Pa}), \\
&c'_{12} = 0.352 \text{ x}10^{11}, && c'_{13} = c'_{23} = 0.639 \text{ x}10^{11} && (\text{Pa}), \\
&c'_{44} = c'_{55} = 0.796 \text{ x}10^{11}, && c'_{66} = 0.509 \text{ x}10^{11} && (\text{Pa}).
\end{aligned}
\tag{S6}
$$

The substrate is treated as an elastic solid with cubic symmetry [1,1,0] using values typical of silicon (diamond cubic structure). The elastic constants (using Voigt notation) are taken as

$$
\begin{aligned}
&c_{11} = c_{22} = 1.944 \text{ x}10^{11}, && c_{33} = 1.657 \text{ x}10^{11} && (\text{Pa}), \\
&c_{12} = 0.352 \text{ x}10^{11}, && c_{13} = c_{23} = 0.639 \text{ x}10^{11} && (\text{Pa}), \\
&c_{44} = c_{55} = 0.796 \text{ x}10^{11}, && c_{66} = 0.509 \text{ x}10^{11} && (\text{Pa}).
\end{aligned}
\tag{S7}
$$

As an axi-symmetric model is being considered, and shear deformation will be encountered in some situations, it is useful to use a transverse isotropic set of properties. This can be achieved in (S7) by selecting $c_{66} = (c_{11} - c_{12})/2 = 0.796 \text{ x}10^{11} \text{ Pa}$. The properties to be used for silicon [110] are then specified by

$$
\boxed{
\begin{aligned}
&c_{11} = c_{22} = 1.944 \text{ x}10^{11}, && c_{33} = 1.657 \text{ x}10^{11} && (\text{Pa}), \\
&c_{12} = 0.352 \text{ x}10^{11}, && c_{13} = c_{23} = 0.639 \text{ x}10^{11} && (\text{Pa}), \\
&c_{44} = c_{55} = c_{66} = 0.796 \text{ x}10^{11} && && (\text{Pa}).
\end{aligned}}
\tag{S8}
$$

Upon inverting the moduli matrix c_{ij} specified by (S8), the values of the components of the compliance matrix (from code using Voigt notation) for silicon [110] are given by

$$
\boxed{
\begin{aligned}
&s_{11} = s_{22} = 5.913618978 \text{ x } 10^{-12}, && s_{33} = 7.684506816 \text{ x } 10^{-12} && (\text{Pa}^{-1}), \\
&s_{12} = -\ 0.3677880572 \text{ x } 10^{-12}, && s_{13} = s_{23} = -2.138675895 \text{ x } 10^{-12} && (\text{Pa}^{-1}), \\
&s_{44} = s_{55} = s_{66} = 12.56281407 \text{ x } 10^{-12} && && (\text{Pa}^{-1}).
\end{aligned}}
\tag{S9}
$$

As the verification procedure will result in very small differences between the analytical model and FEA predictions, it is important to input the compliance data (S9) as accurately as possible. The s_{ij} values given in (S9) are the components of the inverse matrix of the stiffness matrix (S8) to within very small numerical errors and form a consistent set of anisotropic property data for input into both the FEA and analytical models.

S2. Properties of PR (SmSe)

The piezoresistive material (PR) is selected as SmSe with the following elastic properties given in Table 4 of reference [8]:

$$c_{11} = c_{22} = c_{33} = 1.08 \text{ x} 10^{11} \quad \text{(Pa)},$$
$$c_{12} = c_{13} = c_{23} = 0.108 \text{ x} 10^{11} \quad \text{(Pa)}, \qquad \text{(S10)}$$
$$c_{44} = c_{55} = c_{66} = 0.214 \text{ x} 10^{11} \quad \text{(Pa)}.$$

As these properties are not transverse isotropic, the value of c_{66} is changed to the value

$$c_{66} = (c_{11} - c_{12}) / 2 = 0.486 \text{ x} 10^{11} \text{ Pa} .$$

The elastic properties of the PR are then taken as

$$\boxed{\begin{array}{l} c_{11} = c_{22} = c_{33} = 1.08 \text{ x} 10^{11} \quad \text{(Pa)}, \\ c_{12} = c_{13} = c_{23} = 0.108 \text{ x} 10^{11} \quad \text{(Pa)}, \\ c_{44} = c_{55} = 0.214 \text{ x} 10^{11}, \quad c_{66} = 0.486 \text{ x} 10^{11} \quad \text{(Pa)}. \end{array}} \qquad \text{(S11)}$$

Upon inverting, the corresponding compliance values are given by

$$\boxed{\begin{array}{l} s_{11} = s_{22} = s_{33} = \ \ 9.430727023 \text{ x} 10^{-12} \quad (\text{Pa}^{-1}), \\ s_{12} = s_{13} = s_{23} = -0.8573388203 \text{ x} 10^{-12} \quad (\text{Pa}^{-1}), \\ s_{44} = s_{55} = 46.72897196 \text{ x} 10^{-12}, \quad s_{66} = 20.57613169 \text{ x} 10^{-12} \quad (\text{Pa}^{-1}). \end{array}} \qquad \text{(S12)}$$

As the verification procedure will result in very small differences between the analytical model and FEA predictions, it is again important to input the compliance data (S12) as accurately as possible. The s_{ij} values given in (S12) are the components of the inverse matrix of the stiffness matrix (S11) to within very small numerical errors and form a consistent set of property data for input into both the FEA and the analytical model.

S3. Properties of PE (PMN-33%Pt)

When considering the properties of the piezoelectric material (PE), selected as PMN-33%Pt in this case, account must be taken of the way in which the poling direction is modelled. For the analytical model, poling is assumed to be in the x_3 direction, whereas for Abaqus (using an axi-symmetric model) poling is taken in the x_2 direction. The following input data assume that the PE is poled in the vertical direction, i.e. the x_3 direction.

Initially, the assumed properties for PMN-33%Pt are those given in Table II of reference [9] (to be referred to as Table II without the reference number). The poling of the material is in the x_3 direction. The values of particular interest are the elastic compliances s^E_{pq}, the piezoelectric coefficients d_{ip}, and the relative permittivities ε^T_{ij}, where *i* and *j* take the values 1, 2, and 3 and *p* and *q* take the values 1–6, where the following Voigt notation is used to denote component ordering of *pq* values when using the contracted notation:

$$(11, 22, 33, 23, 13, 12).$$

From Table II, the non-zero properties to be used here are given by

$$\begin{aligned} &s_{11}^E = 69.0 \text{ x } 10^{-12} \text{ Pa}^{-1}, \quad s_{22}^E = 69.0 \text{ x } 10^{-12} \text{ Pa}^{-1}, \quad s_{33}^E = 119.6 \text{ x } 10^{-12} \text{ Pa}^{-1}, \\ &s_{12}^E = -11.1 \text{ x } 10^{-12} \text{ Pa}^{-1}, \quad s_{13}^E = -55.7 \text{ x } 10^{-12} \text{ Pa}^{-1}, \quad s_{23}^E = -55.7 \text{ x } 10^{-12} \text{ Pa}^{-1}, \\ &s_{44}^E = 14.5 \text{ x } 10^{-12} \text{ Pa}^{-1}, \quad s_{55}^E = 14.5 \text{ x } 10^{-12} \text{ Pa}^{-1}, \quad s_{66}^E = 15.2 \text{ x } 10^{-12} \text{ Pa}^{-1}, \end{aligned} \tag{S13}$$

$$\begin{aligned} &d_{31} = -1330 \text{ x } 10^{-12} \text{ C/N}, \quad d_{32} = -1330 \text{ x } 10^{-12} \text{ C/N}, \quad d_{33} = 2820 \text{ x } 10^{-12} \text{ C/N}, \\ &d_{15} = 146 \text{ x } 10^{-12} \text{ C/N}, \quad d_{24} = 146 \text{ x } 10^{-12} \text{ C/N}, \end{aligned} \tag{S14}$$

$$\varepsilon_{11}^T = 1600, \quad \varepsilon_{22}^T = 1600, \quad \varepsilon_{33}^T = 8200. \tag{S15}$$

When using Abaqus, it is convenient to input values of the matrix c_{pq}^E, which is the inverse of the compliance matrix s_{pq}^E specified by (S13). It can be seen that the corresponding values of c_{pq}^E are given, from code output using the above values of s_{pq}^E, by

$$\begin{aligned} &c_{11} = 0.8931390564 \text{ x } 10^{11}, \quad c_{22} = 0.8931390564 \text{ x } 10^{11}, \quad c_{33} = 0.8043230628 \text{ x } 10^{11} \quad \text{(Pa)}, \\ &c_{12} = 0.7682951113 \text{ x } 10^{11}, \quad c_{13} = 0.7737615647 \text{ x } 10^{11}, \quad c_{23} = 0.7737615647 \text{ x } 10^{11} \quad \text{(Pa)}, \\ &c_{44} = 0.6896551724 \text{ x } 10^{11}, \quad c_{55} = 0.6896551724 \text{ x } 10^{11}, \quad c_{66} = 0.6578947368 \text{ x } 10^{11} \quad \text{(Pa)}, \end{aligned}$$

so that upon rounding to 3 significant figures,

$$\begin{aligned} &c_{11}^E = 89.3 \text{ GPa}, \quad c_{22}^E = 89.3 \text{ GPa}, \quad c_{33}^E = 80.4 \text{ GPa}, \\ &c_{12}^E = 76.8 \text{ GPa}, \quad c_{13}^E = 77.4 \text{ GPa}, \quad c_{23}^E = 77.4 \text{ GPa}, \\ &c_{44}^E = 69.0 \text{ GPa}, \quad c_{55}^E = 69.0 \text{ GPa}, \quad c_{66}^E = 65.8 \text{ GPa}. \end{aligned} \tag{S16}$$

These property values are the non-zero components of the matrix

$$\begin{bmatrix} c_{11}^E & c_{12}^E & c_{13}^E & 0 & 0 & 0 \\ c_{12}^E & c_{22}^E & c_{23}^E & 0 & 0 & 0 \\ c_{13}^E & c_{23}^E & c_{33}^E & 0 & 0 & 0 \\ 0 & 0 & 0 & c_{44}^E & 0 & 0 \\ 0 & 0 & 0 & 0 & c_{55}^E & 0 \\ 0 & 0 & 0 & 0 & 0 & c_{66}^E \end{bmatrix}.$$

It is useful to provide the values of c_{pq}^E that are given in reference [9] (for Voigt contracted notation and poling in the 3-direction), which are

$$\begin{aligned} &c_{11}^E = 115.0 \text{ GPa (89.3 GPa)}, \quad c_{22}^E = 115.0 \text{ GPa (89.3 GPa)}, \quad c_{33}^E = 103.0 \text{ GPa (80.4 GPa)}, \\ &c_{12}^E = 103 \text{ GPa (76.8 GPa)}, \quad c_{13}^E = 102.0 \text{ GPa (77.4 GPa)}, \quad c_{23}^E = 102.0 \text{ GPa (77.4 GPa)}, \\ &c_{44}^E = 69.0 \text{ GPa (69.0 GPa)}, \quad c_{55}^E = 69.0 \text{ GPa (69.0 GPa)}, \quad c_{66}^E = 66.0 \text{ GPa (65.8 GPa)}. \end{aligned} \tag{S17}$$

The values in brackets are the rounded inverted values derived from the s^E_{pq} values (S13) given in reference [9]. It is clear that the substantial differences in the values of c^E_{pq}, p, q = 1, 2, 3, indicate that the c^E_{pq} given in [9] have not been determined by a reliable inversion procedure. This means that the values for c^E_{pq} and s^E_{pq} given in [9] are not consistent with each other. A study of reference [9] reveals that s^E_{11}, c^E_{11}, c^E_{44} and c^E_{66} are measured values, and that other values of c^E_{pq} and s^E_{pq}, obtained by various calculations, do not form a consistent set of properties.

Analysis of measured data

A detailed analysis will now be given of the measured data provided in reference [9]. Various relationships between elastic constants that are used in [9] are derived in Appendix A. The analysis is restricted to measurements that are given in Table I of this reference, where measured velocities are given and the values of elastic moduli or combinations of them.

As the relationship between these constants and the velocities is not given in reference [9], use is made of the relationship

$$c_{ij} = \rho v^2, \qquad \text{(S18)}$$

where the density $\rho = 8060$ kg/m^3 (see the heading of Table II) and v is the measured velocity. The elastic constants and their associated measured velocities are as follows:

c^D_{33}	4610 m/s	[001]
c^E_{44}	2930 m/s	[001]
c^E_{11}	3792 m/s	[001]
c^E_{66}	2882 m/s	[001]
c^D_{44}	3099 m/s	[001]
$\frac{1}{2}(c^E_{11} + c^E_{12} + 2c^E_{66})$	4727 m/s	[110]
$\frac{1}{2}(c^E_{11} - c^E_{12})$	880 m/s	[110].

In reference [9], the values of electromechanical coupling coefficients and the three elastic compliances s^E_{11}, s^E_{33}, and s^D_{33} are stated to be provided from the measurement of resonant and anti-resonant frequencies. The values of s^E_{11} and s^E_{33} and the coupling constant k_t are required here, and their values are taken on trust directly from Table II so that

$$s^E_{11} = 69.0 \text{ x } 10^{-12} \text{ Pa}^{-1}, \quad s^E_{33} = 119.6 \text{ x } 10^{-12} \text{ Pa}^{-1},$$
$$k_t = 0.64.$$

The piezoelectric constants d_{31} and d_{33} are stated to be measured quantities and their values in Table II are

$$d_{31} = -1330.0 \text{ x } 10^{-12} \text{ C/N}, \quad d_{33} = 2820.0 \text{ x } 10^{-12} \text{ C/N}.$$

Using the above information, which is regarded as being reliable, other properties will now be estimated and compared with the values given in Table II.

Upon applying the measured velocities to the relation (S18) to estimate elastic constants, it can be shown that

$$c_{11}^E = 115.9 \ (115.0) \ \text{GPa}, \quad c_{44}^E = 69.19 \ (69.0) \ \text{GPa}, \quad c_{66}^E = 66.95 \ (66.0) \ \text{GPa}.$$

The values shown in brackets are taken from Table II. Upon calculating the value of $\frac{1}{2}(c_{11}^E + c_{12}^E + 2c_{66}^E)$ from the appropriate measured velocity, it is then possible to calculate the value

$$c_{12}^E = 110.4 \ (103.0) \ \text{GPa}.$$

Upon calculating the value of $\frac{1}{2}(c_{11}^E - c_{12}^E)$ from another measured velocity, the following alternative value is obtained:

$$c_{12}^E = 103.4 \ (103.0) \ \text{GPa}.$$

The latter value will be assumed as it is closer to the value given in Table II. As the value of s_{11}^E is taken from Table II, use can be made of the relation (SA13) to determine the following value for s_{12}^E:

$$s_{12}^E = -11.11 \ (-11.1 \text{ x} 10^{-12}) \ \text{Pa}^{-1}.$$

It is then possible to use the relation (SA14) and the value of s_{33}^E taken from Table II to calculate the following value of s_{13}^E:

$$s_{13}^E = -56.47 \ (-55.7 \text{ x} 10^{-12}) \ \text{Pa}^{-1}.$$

The relations (SA10)$_{2,3}$ may then be used to calculate the following values of c_{13}^E and c_{33}^E:

$$c_{13}^E = 103.56 \ (103.0) \ \text{GPa},$$
$$c_{33}^E = 106.16 \ (103.0) \ \text{GPa}.$$

An alternative value of c_{33}^E can be obtained using equation (1) of reference [9], namely,

$$c_{33}^E = c_{33}^D(1 - k_t^2), \qquad \text{(S19)}$$

which leads to the value

$$c_{33}^E = 101.13 \ (103.0) \ \text{GPa}.$$

The value of c_{33}^E given in Table II is almost the average of the values that can be calculated directly. The recommended approach is to use the value $c_{33}^E = 106.16 \ (103.0) \ \text{GPa}$ resulting from the use of the relation (SA10)$_3$ as this value should also result if the corresponding compliance matrix is inverted.

Upon assembling the assumed and calculated values (obtained from code) of the elastic constants of PMN-33%Pt, the following sets of values should form a consistent set of recommended values that are inverses of one another:

Moduli in Pa:

$$c_{11}^E = 0.1158968678 \text{ x } 10^{12},\quad c_{12}^E = 0.1034135398 \text{ x } 10^{12},\quad c_{13}^E = 0.1035567302 \text{ x } 10^{12},$$

$$c_{33}^E = 0.1061586271 \text{ x } 10^{12},\quad c_{44}^E = 0.6919429400 \text{ x } 10^{11},\quad c_{66}^E = 0.6694574742 \text{ x } 10^{11},$$

so that upon rounding the recommended values are

$$\boldsymbol{c}^E = \begin{bmatrix} 115.90 & 105.41 & 103.56 & 0 & 0 & 0 \\ 105.41 & 115.90 & 103.56 & 0 & 0 & 0 \\ 103.56 & 103.56 & 106.16 & 0 & 0 & 0 \\ 0 & 0 & 0 & 69.194 & 0 & 0 \\ 0 & 0 & 0 & 0 & 69.194 & 0 \\ 0 & 0 & 0 & 0 & 0 & 66.946 \end{bmatrix} \text{GPa}, \qquad \text{(S20)}$$

and the corresponding values from Table II are (note that the recommended values in (S20) differ significantly from those given by (S16), which are obtained by inverting the matrix $\boldsymbol{s}^E$ defined by the values (S13))

$$\boldsymbol{c}^E = \begin{bmatrix} 115.0 & 103.0 & 102.0 & 0 & 0 & 0 \\ 103.0 & 115.0 & 102.0 & 0 & 0 & 0 \\ 102.0 & 102.0 & 103.0 & 0 & 0 & 0 \\ 0 & 0 & 0 & 69.0 & 0 & 0 \\ 0 & 0 & 0 & 0 & 69.0 & 0 \\ 0 & 0 & 0 & 0 & 0 & 66.0 \end{bmatrix} \text{GPa}. \qquad \text{(S21)}$$

Compliances in Pa:

$$s_{11}^E = 0.6900000000 \text{ x } 10^{-10},\quad s_{12}^E = -0.1110684330 \text{ x } 10^{-10},\quad s_{13}^E = -0.5647422326 \text{ x } 10^{-10},$$

$$s_{33}^E = 1.1960000000 \text{ x } 10^{-10},\quad s_{44}^E = 0.1445205872 \text{ x } 10^{-10},\quad s_{66}^E = 0.1493746860 \text{ x } 10^{-10},$$

so that upon rounding, the recommended values are

$$
\boldsymbol{s}^E = \begin{bmatrix} 0.690 & -0.111 & -0.565 & 0 & 0 & 0 \\ -0.111 & 0.690 & -0.565 & 0 & 0 & 0 \\ -0.565 & -0.565 & 1.196 & 0 & 0 & 0 \\ 0 & 0 & 0 & 0.1445 & 0 & 0 \\ 0 & 0 & 0 & 0 & 0.1445 & 0 \\ 0 & 0 & 0 & 0 & 0 & 0.1494 \end{bmatrix} \text{ x } 10^{-10}\,\text{Pa}^{-1} \qquad \text{(S22)}
$$

and the corresponding values from Table II are

$$
\boldsymbol{s}^E = \begin{bmatrix} 0.690 & -0.111 & -0.557 & 0 & 0 & 0 \\ -0.111 & 0.690 & -0.557 & 0 & 0 & 0 \\ -0.557 & -0.557 & 1.196 & 0 & 0 & 0 \\ 0 & 0 & 0 & 0.145 & 0 & 0 \\ 0 & 0 & 0 & 0 & 0.145 & 0 \\ 0 & 0 & 0 & 0 & 0 & 0.152 \end{bmatrix} \text{ x } 10^{-10}\,\text{Pa}^{-1}\,. \qquad \text{(S23)}
$$

While there is little difference between the recommended and Table II values, the former have the advantage that they form a consistent set where $\boldsymbol{c}^E$ and $\boldsymbol{s}^E$ are inverses of one another if a sufficient number of significant figures are used. Furthermore, the recommended values for elastic moduli and compliances given by (S20) and (S22) are directly related to velocity measurements in Table I of reference [9], while this cannot be demonstrated for some of the values given in Table II. It should be noted that the velocity/property relations for the [110] orientation of the crystal assume that $c_{11}^E = c_{22}^E$, $c_{44}^E = c_{55}^E$, $c_{13}^E = c_{23}^E$. Also, it is emphasised that the c_{ij}^E values (S16), obtained by inverting the Table II values given in (S13), differ very significantly from the recommended values appearing in the matrix (S20).

As the recommended properties (S20) are not transverse isotropic, the value of c_{66}^E is changed to the value

$$
c_{66}^E = (c_{11}^E - c_{12}^E)/2 = 5.245 \text{ GPa}\,.
$$

The elastic properties of the PE are then taken as

$$
\boxed{\begin{array}{lll} c_{11}^E = 115.90 \text{ GPa}, & c_{22}^E = 115.90 \text{ GPa}, & c_{33}^E = 106.16 \text{ GPa}, \\ c_{12}^E = 105.41 \text{ GPa}, & c_{13}^E = 103.56 \text{ GPa}, & c_{23}^E = 103.56 \text{ GPa}, \\ c_{44}^E = 69.194 \text{ GPa}, & c_{55}^E = 69.194 \text{ GPa}, & c_{66}^E = 5.245 \text{ GPa}. \end{array}} \qquad \text{(S24)}
$$

Upon using the code (based on Voigt notation) to invert the matrix with components given by (S24), the following values of the compliance matrix s_{ij}^E required by the analytical model are obtained

$$
\boxed{\begin{array}{ll}
s_{11}^E = \;\; 0.7362141751 \text{ x } 10^{-10} \text{ Pa}^{-1}, & s_{22}^E = \;\; 0.7362141751 \text{ x } 10^{-10} \text{ Pa}^{-1}, \\
s_{33}^E = \;\; 1.082241556 \text{ x } 10^{-10} \text{ Pa}^{-1}, & s_{12}^E = -0.2170746714 \text{ x } 10^{-10} \text{ Pa}^{-1}, \\
s_{13}^E = -0.5064250849 \text{ x } 10^{-10} \text{ Pa}^{-1}, & s_{23}^E = -0.5064250849 \text{ x } 10^{-10} \text{ Pa}^{-1}, \\
s_{44}^E = \;\; 0.1445212013 \text{ x } 10^{-10} \text{ Pa}^{-1}, & s_{55}^E = \;\; 0.1445212013 \text{ x } 10^{-10} \text{ Pa}^{-1}, \\
\multicolumn{2}{c}{s_{66}^E = \;\; 1.906577693 \text{ x } 10^{-10} \text{ Pa}^{-1}.}
\end{array}} \qquad \text{(S25)}
$$

Piezoelectric constants

The matrix ***d***, defined using the contracted Voigt notation needed for the analytical model, is specified by

$$
\begin{bmatrix} d_{11} & d_{12} & d_{13} & d_{14} & d_{15} & d_{16} \\ d_{21} & d_{22} & d_{23} & d_{24} & d_{25} & d_{26} \\ d_{31} & d_{32} & d_{33} & d_{34} & d_{35} & d_{36} \end{bmatrix} \equiv \begin{bmatrix} 0 & 0 & 0 & 0 & 146 & 0 \\ 0 & 0 & 0 & 146 & 0 & 0 \\ -1330 & -1330 & 2820 & 0 & 0 & 0 \end{bmatrix} \text{ x } 10^{-12} \text{ C / N}.
$$

The corresponding piezoelectric coefficients d_{ijk} are obtained from the contracted d_{ip} values, based on Voigt notation, using the following identification:

$$
\begin{bmatrix} d_{11} & d_{21} & d_{31} \\ d_{12} & d_{22} & d_{32} \\ d_{13} & d_{23} & d_{33} \\ d_{14} & d_{24} & d_{34} \\ d_{15} & d_{25} & d_{35} \\ d_{16} & d_{26} & d_{36} \end{bmatrix} \equiv \begin{bmatrix} d_{111} & d_{211} & d_{311} \\ d_{122} & d_{222} & d_{322} \\ d_{133} & d_{233} & d_{333} \\ 2d_{123} & 2d_{223} & 2d_{323} \\ 2d_{113} & 2d_{213} & 2d_{313} \\ 2d_{112} & 2d_{212} & 2d_{312} \end{bmatrix}.
$$

Dielectric constants

The relative permittivity values ε_{ij}^T, $i, j = 1, 2, 3$, used for PMN-33%Pt are

$$
\varepsilon_{11}^T = \varepsilon_{22}^T = 1600, \;\; \varepsilon_{33}^T = 8200, \;\; \text{(poling in the 3-direction).}
$$

The value of the dielectric constant for free space is taken as

$$
\varepsilon_0 = 8.854 \text{ x } 10^{-12} \;\; \text{F/m},
$$

so that the dielectric constants needed by Abaqus and the analytical model are given by

$$
\varepsilon_{11}^T \varepsilon_0 = \varepsilon_{22}^T \varepsilon_0 = 14.1664 \text{ x } 10^{-9} \;\; \text{F/m}, \qquad \varepsilon_{33}^T \varepsilon_0 = 72.6028 \text{ x } 10^{-9} \;\; \text{F/m}.
$$

Appendix SA: Derivations of key relations used in reference [9]

The stress–strain relations are

$$\begin{aligned} T_{11} &= c_{11}^{E}S_{11} + c_{12}^{E}S_{22} + c_{13}^{E}S_{33}\,, \\ T_{22} &= c_{12}^{E}S_{11} + c_{22}^{E}S_{22} + c_{23}^{E}S_{33}\,, \\ T_{33} &= c_{13}^{E}S_{11} + c_{23}^{E}S_{22} + c_{33}^{E}S_{33}\,. \end{aligned} \tag{SA1}$$

Since $c_{11}^{E} = c_{22}^{E}$ and $c_{13}^{E} = c_{23}^{E}$, it follows that

$$\begin{aligned} T_{11} &= c_{11}^{E}S_{11} + c_{12}^{E}S_{22} + c_{13}^{E}S_{33}\,, \\ T_{22} &= c_{12}^{E}S_{11} + c_{11}^{E}S_{22} + c_{13}^{E}S_{33}\,, \\ T_{33} &= c_{13}^{E}S_{11} + c_{13}^{E}S_{22} + c_{33}^{E}S_{33}\,. \end{aligned} \tag{SA2}$$

This leads to a solution such that $T_{11} = T_{22}$ and $S_{11} = S_{22}$ where the strains S_{11} and S_{33} satisfy

$$\begin{aligned} T_{11} &= \left(c_{11}^{E} + c_{12}^{E}\right)S_{11} + c_{13}^{E}S_{33}\,, \\ T_{33} &= 2c_{13}^{E}S_{11} + c_{33}^{E}S_{33}\,. \end{aligned} \tag{SA3}$$

It then follows that

$$S_{11} = \frac{c_{33}^{E}T_{11} - c_{13}^{E}T_{33}}{\left(c_{11}^{E} + c_{12}^{E}\right)c_{33}^{E} - 2(c_{13}^{E})^{2}}\,, \quad S_{33} = \frac{\left(c_{11}^{E} + c_{12}^{E}\right)T_{33} - 2c_{13}^{E}T_{11}}{\left(c_{11}^{E} + c_{12}^{E}\right)c_{33}^{E} - 2(c_{13}^{E})^{2}}\,. \tag{SA4}$$

The inverse form of (SA3) is

$$\begin{aligned} S_{11} &= \left(s_{11}^{E} + s_{12}^{E}\right)T_{11} + s_{13}^{E}T_{33}\,, \\ S_{33} &= 2s_{13}^{E}T_{11} + s_{33}^{E}T_{33}\,, \end{aligned} \tag{SA5}$$

and it follows that

$$\begin{aligned} \left(s_{11}^{E} + s_{12}^{E}\right)T_{11} + s_{13}^{E}T_{33} &= \frac{c_{33}^{E}T_{11} - c_{13}^{E}T_{33}}{\left(c_{11}^{E} + c_{12}^{E}\right)c_{33}^{E} - 2(c_{13}^{E})^{2}}\,, \\ 2s_{13}^{E}T_{11} + s_{33}^{E}T_{33} &= \frac{\left(c_{11}^{E} + c_{12}^{E}\right)T_{33} - 2c_{13}^{E}T_{11}}{\left(c_{11}^{E} + c_{12}^{E}\right)c_{33}^{E} - 2(c_{13}^{E})^{2}}\,. \end{aligned} \tag{SA6}$$

As these relations must be satisfied for all values of T_{11} and T_{33}

$$\begin{aligned} s_{11}^{E} + s_{12}^{E} &= \frac{c_{33}^{E}}{\left(c_{11}^{E} + c_{12}^{E}\right)c_{33}^{E} - 2(c_{13}^{E})^{2}}\,, \quad s_{13}^{E} = -\frac{c_{13}^{E}}{\left(c_{11}^{E} + c_{12}^{E}\right)c_{33}^{E} - 2(c_{13}^{E})^{2}}\,, \\ s_{33}^{E} &= \frac{c_{11}^{E} + c_{12}^{E}}{\left(c_{11}^{E} + c_{12}^{E}\right)c_{33}^{E} - 2(c_{13}^{E})^{2}}\,. \end{aligned} \tag{SA7}$$

Alternatively, since from (SA5), namely,

$$S_{11} = \left(s_{11}^E + s_{12}^E\right) T_{11} + s_{13}^E T_{33} \,,$$
$$S_{33} = 2 s_{13}^E T_{11} + s_{33}^E T_{33} \,,$$

it follows that

$$T_{11} = \frac{s_{33}^E S_{11} - s_{13}^E S_{33}}{s_{33}^E \left(s_{11}^E + s_{12}^E\right) - 2(s_{13}^E)^2} \,, \qquad T_{33} = \frac{\left(s_{11}^E + s_{12}^E\right) S_{33} - 2 s_{13}^E S_{11}}{s_{33}^E \left(s_{11}^E + s_{12}^E\right) - 2(s_{13}^E)^2} \,. \tag{SA8}$$

Upon comparing with (SA3), namely,

$$T_{11} = \left(c_{11}^E + c_{12}^E\right) S_{11} + c_{13}^E S_{33} \,,$$
$$T_{33} = 2 c_{13}^E S_{11} + c_{33}^E S_{33} \,,$$

it is clear that

$$\begin{aligned} \left(c_{11}^E + c_{12}^E\right) S_{11} + c_{13}^E S_{33} &= \frac{s_{33}^E S_{11} - s_{13}^E S_{33}}{s_{33}^E \left(s_{11}^E + s_{12}^E\right) - 2(s_{13}^E)^2} \,, \\ 2 c_{13}^E S_{11} + c_{33}^E S_{33} &= \frac{\left(s_{11}^E + s_{12}^E\right) S_{33} - 2 s_{13}^E S_{11}}{s_{33}^E \left(s_{11}^E + s_{12}^E\right) - 2(s_{13}^E)^2} \,. \end{aligned} \tag{SA9}$$

It then follows that

$$\begin{aligned} c_{11}^E + c_{12}^E &= \frac{s_{33}^E}{s_{33}^E \left(s_{11}^E + s_{12}^E\right) - 2(s_{13}^E)^2} \,, \\ c_{13}^E &= - \frac{s_{13}^E}{s_{33}^E \left(s_{11}^E + s_{12}^E\right) - 2(s_{13}^E)^2} \,, \qquad \text{(see eqns.(2), (7) in ref. [9])}, \\ c_{33}^E &= \frac{s_{11}^E + s_{12}^E}{s_{33}^E \left(s_{11}^E + s_{12}^E\right) - 2(s_{13}^E)^2} \,. \end{aligned} \tag{SA10}$$

From the first two relations of (SA2)

$$T_{11} - T_{22} = \left(c_{11}^E - c_{12}^E\right)\left(S_{11} - S_{22}\right) . \tag{SA11}$$

The inverse relation has the form

$$S_{11} - S_{22} = \left(s_{11}^E - s_{12}^E\right)\left(T_{11} - T_{22}\right) . \tag{SA12}$$

It then follows from (SA11) and (SA12) that

$$c_{11}^E - c_{12}^E = \frac{1}{s_{11}^E - s_{12}^E} \quad \text{(see eqn.(3) in ref. [9])} \,. \tag{SA13}$$

Since from (SA10)

$$c_{11}^E + c_{12}^E = \frac{s_{33}^E}{s_{33}^E \left(s_{11}^E + s_{12}^E\right) - 2(s_{13}^E)^2} \,,$$

it follows that

$$\frac{s_{33}^E}{c_{11}^E + c_{12}^E} = s_{33}^E \left(s_{11}^E + s_{12}^E \right) - 2(s_{13}^E)^2 \ ,$$

and upon using (SA13), it follows that

$$\begin{aligned} 2(s_{13}^E)^2 &= s_{33}^E \left(s_{11}^E + s_{12}^E - \frac{1}{c_{11}^E + c_{12}^E} \right) = s_{33}^E \frac{\left(s_{11}^E + s_{12}^E\right)\left(c_{11}^E + c_{12}^E\right) - 1}{c_{11}^E + c_{12}^E} \\ &= s_{33}^E \frac{\left(s_{11}^E + s_{12}^E\right)\left(c_{11}^E + c_{12}^E\right) - \left(s_{11}^E - s_{12}^E\right)\left(c_{11}^E - c_{12}^E\right)}{c_{11}^E + c_{12}^E} \\ &= 2 s_{33}^E \frac{c_{11}^E s_{12}^E + c_{12}^E s_{11}^E}{c_{11}^E + c_{12}^E} \ . \end{aligned}$$

Thus, upon choosing the appropriate sign when taking the square root,

$$s_{13}^E = -\sqrt{s_{33}^E \frac{c_{11}^E s_{12}^E + c_{12}^E s_{11}^E}{c_{11}^E + c_{12}^E}} \qquad \text{(see eqn.(3) in ref. [9])} \ . \qquad \text{(SA14)}$$
